# Linguistic Analysis of the Preschool Five Minute Speech Sample: *What* the Parents of Preschool Children with Early Signs of ADHD Say and *How* They Say It?

**DOI:** 10.1371/journal.pone.0106231

**Published:** 2014-09-03

**Authors:** Elvira Perez, Melody Turner, Anthony Fisher, Joanna Lockwood, David Daley

**Affiliations:** 1 University of Nottingham, School of Medicine, Nottingham, England, United Kingdom; 2 University of Nottingham, School of English, Nottingham, England, United Kingdom; University Children's Hospital Tuebingen, Germany

## Abstract

A linguistic analysis was performed on the Preschool Five Minute Speech Sample (PFMSS) of 42 parents. PFMSS is a validated measure for Expressed Emotion (EE) to assess parent-child relationship. Half of these parents (n = 21, clinical group) had preschool children with early symptoms of attention deficit hyperactivity disorder (ADHD), the rest had typically developing children. Early symptoms of ADHD were identified with the Werry-Weiss Peters Rating Scale. The linguistic component of the PFMSS was analysed with keyword and linguistic pattern identification. The results of these two complementary analyses (i.e., EE and linguistic analysis) provided relevant recommendations that may improve the efficacy of psychological treatment for ADHD such as parenting interventions. We discuss the practical implications of these findings.

## Introduction

Attention deficit hyperactivity disorder (ADHD) is a common disorder that affects individuals across the lifespan and it is most frequently diagnosed during the school years [1]. The UK National Institute for Health and Clinical Excellence (NICE) estimated that about 210,000 children aged 5–18 years in England and Wales are affected by ADHD [2] and its core symptoms of hyperactivity, impulsivity, and inattention. The impact of ADHD on self-esteem, interpersonal relationships, and academic achievement is also recognised [3], [4]. In the long term, ADHD has also been associated with a significant risk of mental illness and delinquency [5], lower health-related quality of life in all psychosocial areas, and those with comorbidities such as oppositional defiant conduct, internalising, and learning disorders have even greater deficits [6]. In addition to its impact on the daily lives of children, ADHD impacts the health and functioning of families, schools, and the community, creating a substantial burden on families as well as health, social care, and criminal justice systems [7]. Multimodal approaches are recommended for the treatment for ADHD [8], which normally begins during school years. There are multiple evidence-based treatment options, including behaviour therapy and medication. Pharmacological treatments are efficacious [9] and are widely used, however, there are a number of limitations that clinicians, commissioners and service providers should take into account. For example, in a recent systematic review on non-pharmacological interventions for ADHD, Sonuga-Barke and colleagues [10] pointed out that pharmacological treatment efficacy outcomes such as normalisation rates are rare [9]; long-term effectiveness remains to be established [11]; adverse effects on sleep, appetite, and growth, although rarely serious, are common [12]; and some parents and clinicians have reservations about medication use [13] as drug treatment is not recommended for preschool children.

Parents are offered training/education programmes as a first-line treatment [2] and a variety of non-pharmacological interventions are available to treat ADHD symptoms. Evidence for their efficacy has been supported in several systematic reviews and meta-analyses [10], [14]–[18]. Results from a recent meta-analysis [10], however, has shown that the efficacy of behavioural interventions was largely reduced when analysing data from informants who were *probably blind* to treatment status, rather than informants who were involved in treatment delivery (*most proximal*) such as parents. In contrast to the lack of blinded evidence of ADHD symptom reduction, behavioural interventions can have a broader positive impact on a range of other outcomes such as improved parenting (e.g., more positive parenting self-concept [19] and reduced childhood conduct problems [20]. Other studies have shown that the efficacy of well-designed parenting programmes is mediated, however, by successful delivery, implementation, and parent engagement [21]. Parents are primary figures in their children's environment and adverse familial environments are common in ADHD families [22], [23]. Whilst unlikely to play a causal role, parenting may be a source of environmental risk involved in the manifestation of ADHD in children with genetic vulnerability [24]. Genetic studies have indicated that ADHD is a highly complex and heterogeneous genetic condition, with multiple genes of very small effect implicated to different degrees across affected individuals. Genes interact with environments so that increased liability for a disorder associated with a gene may be seen more for individuals exposed to particular environmental risks [25]. Accordingly, high levels of conflict and criticism in the families of children with ADHD may moderate the genetic expression for ADHD severity and comorbid conduct problems [26]. Parenting may be related to the development and maintenance of co-morbid disruptive behaviours that commonly occur in children with ADHD [22], [23]. There is growing evidence of the significant relationship between parenting and impairments in domains such as academic and social functioning [22] and neuropsychological functioning [27]. Parenting interventions may also be a component of treatment that could be able to target functional impairments often associated with ADHD including, neuropsychological deficits, academic underachievement, maladaptive social and peer functioning, and disruptive behaviour [28].

Parents' interpretation of their child's behaviour may be especially relevant due to the centrality of parent-child relationships for children's optimal attachment and continuous development [29]–[32]. Parental perceptions of children's ADHD symptomatic behaviour can influence and feed the quality of parent-child relationships. Accordingly, parents' interpretations can aggravate or lessen the impact of ADHD symptoms on parents and parent-child relationships. In a recent study Lench *et al*. [33] assessed the impact of positive interpretations by comparing parents' perceptions of 7–12 years old children with a diagnosis of ADHD and their reports of stressful interactions in three groups of parents: (1) parents of children identified as having ADHD and who use that label, (2) parents of children identified as having ADHD who view their child's ADHD as indicating special abilities (i.e., Indigo Children), and (3) controls (no ADHD diagnosis). Results revealed that parents who perceived symptomatic behaviour as a sign of positive characteristics reported less frequent negative experiences with their child and less intense negative emotions during those experiences. They also view their children as more self-efficacious and as more likely to have a positive future.

For adherence to ADHD treatment to proceed most effectively, professional and lay perceptions also must be reconciled so that the professional expertise of clinicians and researchers, and the personal experiences and beliefs of families jointly determine acceptable and appropriate treatment options [34]. Prior research has documented the importance of understanding *both* physicians' and families' explanatory models of illness as inputs to medical decision making [35]. In order to identify ecological valid outcome measures for the design of effective parenting programmes it is important to comprehend parents' understanding of their child's ADHD early symptoms. Previous studies combining both quantitative and qualitative methodological approaches for the study of ADHD support the need for detail and on-going investigation on how to improve parenting interventions. For example, Bussing *et al*. [36] explored the parental self-care strategies used by the carers of 266 children with ADHD with both questionnaires and open-ended questions. Quantitative findings showed that behaviour modification was the most common strategy amongst these parents, while qualitative analysis pointed out that these parents tried to change their disciplinary action within three domains including changes related to the prevention of disciplinary problems (e.g., time-outs), privilege removal, and parental coping associated with disciplinary problems (e.g., control own emotions, become less judgemental and tolerant and develop more appropriate expectations). Parents' interpretations of their child's ADHD behaviour and the subsequent strategies developed by these parents contain valuable experiential knowledge precious for improving current parenting interventions specifically designed to improve the mental health of both children and carers. Parenting interventions have the potential to also influence parents' perceptions of their child's early ADHD symptoms and therefore maximise treatment efficacy. For example, recognising that it is not the child's fault, may help parents to cope more effectively with challenging behaviours (e.g., fidgeting instead of sitting quietly).

Parents' perspectives have been studied to inform healthcare practitioners and researchers, for example, Hermasen and colleagues [37] conducted a pure phenomenological qualitative study to explore the issues five mothers with children diagnosed with ADHD faced while their children received a non-pharmaceutical intervention (i.e., chiropractic care and the use of an interactive metronome). The themes that emerged were again ecologically relevant to inform about the struggles that carers perceived. Emerging themes from the semi-structured interviews included: medication as their last resort, the importance of family structure, lack of support from the health and educational system and patient satisfaction. Finally, Fiks *et al*. [34] applied freelisting, a standard approach used in anthropology which consists of generating a list of terms to describe perceptions or definitions of a domain. In this mixed methods approach the shared cultural model for the domain is determined from the word choice, order of recall, and modifying terms that are used. Paediatricians and parents of ADHD children systematically explored and shared divergent notions of ADHD. Word lists suggested differing needs and goals for clinicians and parents valuable to foster patient-centred care.

### Parental Expressed Emotion

The *Five Minutes Speech Sample* (FMSS) [38] was originally developed as a brief measure to assess expressed emotion (EE) in relatives of adult psychiatric patients. EE is considered a measure of the patient-relative relationship and is a highly valid and reliable predictor of poor clinical outcomes among patients with major psychopathology [39]. The FMSS has been used increasingly to examine the emotional quality of relationships in families with adolescents and children [40], [41]. Associations and a range of childhood psychiatric diagnoses, medical conditions and behaviour problems have also been documented [42]. Studies have consistently found that high parental expressed emotion represents a negative family process that is different from other types of family distress and dysfunction [43].

Negative parental EE has been associated with higher levels of ADHD symptoms and conduct problems in children [44]. Likewise, parents express more negative emotions towards their child with an ADHD diagnosis compared with controls [45], [46] or unaffected siblings [47]. The relationship between EE and ADHD is still not clear but it is accepted that negative parenting starts as a reaction to the ADHD behaviour in children. Subsequently, negative parenting increases the chances of developing oppositional behaviours in children with ADHD. This in turn may lead to negative parenting, causing a negative spiral of child behaviour affective parenting and so on. However, a recent re-analysis of Cartwright et al [48] has shown that family characteristics play a role in driving negative EE. This study demonstrates that maternal depression and variation in child comorbid symptoms, especially in relation to oppositional and conduct problems rather than ADHD, also influence EE components. These results highlight (1) the value of interventions targeting broader family/siblings and parental mental health in the management of ADHD and (2) the putative role that the family environment may have in ADHD.

In a recent study Richard and colleagues [49] investigated the cross-sectional and longitudinal relationships between conduct problems in children with ADHD and parental EE. Their results showed a negative cross-sectional association between EE (maternal warmth and criticism) and child ADHD symptom severity, and child oppositional and conduct problems. EE was not significantly correlated over time (six years), suggesting that EE is not a predictive measure but rather a snapshot of a momentary state sensitive to contextual and developmental factors. From a developmental psychopathology perspective, each developmental stage comes with specific vulnerabilities that change over time. Parents of preschool children with ADHD may experience different challenges and expectations than those with older children. Because of its sensitivity to developmental phases, EE has the potential to capture parents' perspectives and provide a useful tool to understand parent's perceptions with the objective to maximise the relevancy of early interventions.

The current study applies knowledge from health humanities, such as applied linguistics, to bring new insights and resources for practitioners and researchers interested in developing relevant parenting skills and outcome measures for the assessment of parenting interventions. To our knowledge, linguistic methods have not been used to date. We hypothesise that a deeper understanding of (1) how parents organise language to describe their child's behaviour and (2) how parents linguistically construct images of their child will inform efforts to further understand parents' perceptions, maximise the relevancy and effectiveness of parenting interventions and consequently, improve treatment outcomes and child functioning. To test this hypothesis, the linguistic patterns emerging from the analysis of the speech samples from parents whose pre-school children exhibit early ADHD symptoms and those from matched controls will be compared.

## Methods

### Setting

Recruitment for the clinical sample was made through radio adverts, posters and referrals from family support workers and healthcare practitioners in Nottingham (UK) and was embedded within a study exploring children at risk of developing ADHD. Recruitment for the control group was made by advertising the study in 40 randomly selected nurseries in Nottingham.

This study has been approved by clinical and non-clinical Medical Research Committee at Nottingham University. Written informed consent was obtained from all participants. All written documentation, including consent forms, will be stored securely in locked filing cabinets at the Division of Psychiatry and Applied Psychology at the Institute of Mental Health, Nottingham University. All participants were assigned an ID number during preparation of transcripts and only members of the research team had access to the participant's contact details. All data has been stored and will be kept for five years in locked filing cabinets prior to being destroyed securely.

### Sample

We conducted individual interviews with a purposive sample of 42 parents of children between 2 years 9 months and 4 years 9 months. Half of these parents (n = 21) had children who scored ≥20 and the other half (n = 21) scored <20 in the WWP activity questionnaire which is a valid tool to discriminate between hyperactive and normally developing children. Participants were informed that the interviews would be audiotaped and used for research purposes. All participants were given the opportunity to decline participation or terminate the interview at any point. The 2.9 to 4.6 year old range was chosen to match that preschool age when ADHD symptoms start to be noticeable and early prevention of later ADHD symptoms. Note ADHD guidelines [2] recommend diagnosis and pharmacological treatment for children ≥6 years of age.

### Data collection

In the current study we used an adapted version of the FMSS, the Preschool Five Minutes Speech Sample (PFMSS) [50]. To obtain the Preschool FMSS parents were asked to talk for five minutes and express their thoughts and feeling about their preschool child, what kind of person the child is and how they get along with the child. Monologues were recorded without interruptions. If the parent stopped speaking before the five minutes had elapsed the researcher would wait for 30 seconds and then prompt the parent by saying “Please, tell me anything about (child's name) for a few more minutes”. Samples from both groups (clinical and controls) were audio recorded with an *Olympus* digital voice recorder WS-450s and transcribed by three of the authors (M. T., E. P. and J.L.).When recordings were made over the phone researchers used a *Re-Tell* telephone recording connector and a *Polycom SoundPoint IP 331* digital telephone. Content analysis for all transcriptions was done according to Wearder's rating scale [51] to assess the emotional climate of the parent-child relationship. PFMSS yields to three global ratings: initial statement, relationship, and warmth as well as frequency counts of critical comments and positive comments. The PFMSS has demonstrated good code-recode and inter-rater reliability, and adequate test-retest reliability [50]. High or negative EE results from at least one negative or low global category and more critical comments than positive comments. All data collectors were trained using the same collection and coding manual [51].

Transcriptions for all 42 FMSS were completed by M.T. In addition, two other trained coders (E.P. and J.L.) independently transcribed and coded 20% of the audio samples randomly selected to assess inter-rater reliability. Coders E.P. and J.L. were blind to the ADHD status of the children and parent's demographic data. Additional editing was required by J.L. to solve transcription difficulties from participants with strong regional accents.

Participants also completed a child's activity questionnaire, the *Werry-Weiss Peters Rating Scale* (WWP) [52]. This a 27-item questionnaire which identifies the top 15-18% of the population using a score of 20 as cut off [53]. The psychometric properties of the WWP have been reviewed by [54] who reported the discrimination between hyperactive and normally developing children to be good. The inter-parent agreement has also been found to be good (r = 0.82) [55]. The WWP takes approximately five minutes to administer and has been shown to have high levels of internal consistency, to correlate with other measures of hyperactivity, and to identify children who have activity problems five years later [56]. This questionnaire includes questions such as ‘During meals is the child up and down at the table?’ giving three possible answers: ‘No, or hardly ever’, ‘Yes, fairly often’ and ‘Yes, very often’.

To ensure that both clinical and control groups were matched and to avoid confound factors, parents completed a *demographics* questionnaire that included questions on age, occupation, income, education, relationship status and the child's health a year after birth.

### Statistical analysis

SPSS (V.21) software was used to analyse the data. A one-way random Intraclass Correlation Coefficient (ICC) was carried out to check reliability between PFMSS coders. Transcription reliability was calculated as the percentage of agreement within the 20% of the audio samples randomly selected for this purpose using the following formula: Number of linguistic units scored identically divided by total number of units scored and multiplied by 100. A chi square (*X^2^*) statistic was used to calculate whether distributions of categorical variables differed between groups. The Kolmogorov-Smirnov test was used to determine which t-test to use depending on how data was distributed. The Kolmogorov-Smirnov test showed that all scores were normally distributed (*p*>0.05) apart from EE total (*p* = 0.00) which required non-parametric statistics (Spearman to calculate correlations and Mann-Whitney U for t-tests). As such Independent Samples T-tests were used to look for significant differences between the two groups. The significance level in this experiment was *p*<0.05. Multivariate analysis of variance controlling for covariates was used to control for demographic differences between participants. Person's correlations were conducted to further explore the associations between child's active behaviour, expressed emotion and socioeconomic status. Cohen's [57] conventions were used to interpret effect size.

Keyword lists were generated using *Wordsmith Tools* and calculated using log-likelihood with a *p* value of 0.00001.

### Data analysis

To interpret the PFMSS we adopted a mixed quantitative and qualitative design. Content analysis or *what* parents said about their children was assessed by measuring parental expressed emotion (EE) which involved quantitative (e.g., counts of positive/negative comments) and qualitative methods (e.g., identifying statements that expressed warmth or concern). To examine *how* parents described their children we used corpus-assisted discourse analysis [58] such as keyword and linguistic pattern identification. Corpus-assisted discourse analysis leads to a more evidence-based approach to uses of language in different contexts and has been facilitated by the development of computer technology and powerful software. The possibilities for corpus research in health care have been relatively under-explored. Some recent studies [59], [60], [61], [62], [63] have been successful in exploring how a combined qualitative and quantitative methodology, drawing on tools traditionally used for corpus analysis, can enhance our understanding of a particular context. Keyword lists were generated using *Wordsmith Tools* [64], a digital text analysis software package commonly used in corpus linguistics research. Keywords are those items which occur more frequently in the dataset than they do in a larger reference corpus, to an extent that is statistically significant. Thus, keyword lists differ from word lists in that they provide a measure of *saliency* rather than simple frequency ([65] p. 125). For this study the reference corpus used was the 10 million word spoken sub-corpus of the British National Corpus [66], and the keyword lists were calculated using log-likelihood with a *p* value of 0.00001.

Once the keyword lists were generated the items in the lists for the two datasets were examined for patterns or tendencies towards particular semantic fields. A relatively high proportion of potentially evaluative words were identified, and so these items were separated into broadly positive and negative categories.

Frequency lists offer an overview of the number of times any word is used in any given text (corpus) relative to other words. The software can also present all occurrences of any key or important word in the text and the language occurring before and after it (i.e., concordance). Here, we can see the context in which words are used. Word frequency lists and concordances can be used as a diagnostic tool to achieve a baseline for determining *what the text is about*, its patterns and regularities of meaning or semantic prosody, and can underpin and support claims of discourse analysis [61].

The transcripts of the forty-two PFMSS were edited to remove interviewer questions, participant IDs, occasional prompts and filled silences before being converted into a text-file (27,892 words). Our analysis involved examining the words and phrases used by parents when describing their preschool children. Using the textual diagnosis as a guide to salient language, the transcriptions were read several times and annotated by the research team to identify patterns and features of discourse that differed between the PFMSS of the control and the clinical sample. The research team comprised three psychologists, a mature medical student and an applied linguist with a research profile in health discourse analysis.

## Results

### Socioeconomic Status

A total of 42 parents were enrolled in the study. In order to assess how similar both groups were and how generalizable our findings are, variability in socioeconomic status (SES) between the two groups was assessed. There is evidence to support that SES has the potential to heavily influence parent-child relationship [67], [68]. Purposive sampling resulted in both parent groups being evenly matched according to age, gender, ethnicity, number of siblings, marital status, and perinatal factors such as type of birth (see Table 1).

**Table 1:** Demographic characteristic of the participants.

	Control group (n = 21)		Clinical group (n = 21)	
**Age**	18–28	1 (4.8%)	18–28	10 (47.6%)
	29–39	13 (61.9%)	29–39	9 (42.9%)
	40–50	6 (28.6%)	40–50	2 (9.5%)
	51–60	1 (4.8%)	51–60	0
**Gender**	Male	16 (76.2%)	Male	19 (90.5%)
	Female	5 (23.8%)	Female	2 (9.5%)
**Ethnicity**	White	19 (90.5%)	White	16 (76.2%)
	Mixed-race	0	Mixed- race	3 (14.3%)
	Afro-Caribbean	0	Afro-Caribbean	2 (9.5%)
	Bangladeshi/Indian	1 (4.8%)	Bangladeshi/Indian	0
	Other	1 (4.8%)	Other	0
**Number of Siblings**	0	4 (19.1%)	0	5 (23.8%)
	1	14 (66.7%)	1	5 (23.8%)
	2	3 (14.3%)	2	6 (28.6%)
	3+	0	3+	5 (23.8%)
**Marital Status**	Separated/Divorced	1 (4.8%)	Separated/Divorced	0
	Married/Living with partner	19 (90.4%)	Married/Living with partner	14 (66.7%)
	Single	1 (4.8%)	Single	7 (33.3%)
**Type of Birth**	Normal	13 (61.9%)	Normal	17 (80.9%)
	Section	5 (23.8%)	Section	3 (14.3%)
	Ventouse	2 (9.5%)	Ventouse	0
	Forceps	1 (4.8%)	Forceps	1 (4.8%)
**Weekly Income**	£200 or below	2 (9.5%)	£200 or below	11 (52.4%)
	£201–250	4 (19%)	£201–250	3 (14.3%)
	£251–300	16 (76.2%)	£251–300	5 (23.8%)
	£300 or below	0	£300 or below	2 (9.5%)
**Educational Level**	University	8 (38.1%)	University	2 (9.5%)
	Postgraduate	11 (52.4%)	Postgraduate	0
	College	2 (9.5%)	College	2 (9.5%)
	NVQs	0	NVQs	7 (33.3%)
	GCSE	0	GCSE	4 (19%)
	No qualification	0	No qualification	6 (28.6%)

The difference between the control and clinical groups' functional income, however, was statistically significant (*X* ^2^ (3) = 10.467; *p*<.05). While most of the clinical sample (n = 11) had a weekly functional income of £200 or below, the control group (n = 15) had a weekly income of £301 or above. Efforts to recruit parents of lower income for the control group were made by promoting recruitment within nurseries from less affluent neighbourhoods, unfortunately, recruitment figures were extremely low when compared to nurseries located in more affluent areas. Regarding educational level, results showed that the difference between the control and clinical groups in terms of education was also statistically significant (*X* ^2^ (5) = 31.600; *p*<0.001). Most parents from the control (n = 11) group had a professional or postgraduate degree, contrasting with the clinical group, in which a high proportion of participants (n = 6) left school at 16 with no qualifications.

There was a tendency for the clinical group to have larger number of siblings (M = 1.762) than the control (M = 0.952) but this difference was not significant (*p*>.05).

### Werry-Weis Peters (WWP) Rating Scale

The WWP Scores of the clinical group (M = 41.714; SD = 8.574) were significantly higher than those of the control group (M = 8.62; SD 4.975; *t* (20) = 22.295, p = 0.000; Cohen's d = 4.721). This result indicated that the study correctly categorised the preschool children as clinically active or showing typically developing levels of activity. When income and education level differences between groups were controlled, analysis of variance for multiple dependent variables showed that neither education level nor income had an effect on WWP scorings (*p*>0.05).

### Expressed Emotion

The components of EE are a rating of warmth, the initial statement the parent makes about the preschool child, and a rating of their relationship. Each component is rated either 1 (positive), 2 (neutral), or 3 (negative). The median EE total for the control group (M = 5.762, SD = 1.300) was lower than the mean EE total for the clinical group (M = 8.619, SD = 0.669). A non-parametric Mann-Whitney U test showed this difference to be significant: U = 14.000; exact *p* = 0.01 (two-tailed). EE was higher for the clinical group indicating that generally those parents expressed a more negative initial statement, showed less warmth, and a worse relationship with their child. This result confirmed that the groups were correctly categorised according to their different emotional relationships with their children. When income and education level differences between groups were controlled as covariates, analysis of variance for multiple dependent variables showed the education level had an effect on EE ratings (F = 5.42 (3, 42), *p* = 0.02), however, income did not affect EE scorings (*p*>0.05).

The positive comments of the clinical group (M = 3.1429; SD 1.681) were significantly lower than those of the control group (M = 9.7619; SD =  3.72699; *t* (20) = 8.563, *p* = .000; Cohen's *d* = 2.891). This result showed that parents of preschool children with early symptoms of ADHD were more likely to give their child less positive comments than if their child was typically developing. The negative comments of the clinical group (M = 8.9048; SD = 4.20600) were significantly higher than those of the control group (M = 2.0000; SD = 2.21359; *t* (20) = 9.702, p = .000; Cohen's *d* = 3.361. This result showed that parents whose children had early symptoms of ADHD were more likely to say negative comments to their child than parents with typically developing children. Parents of preschool children with early ADHD symptoms were about three times as likely to say something negative than a positive comment.

Inter-rater reliability was calculated to measure the reliability between all three coders. Results showed a high correlation of 0.97 *p<0.001* CI 0.877–0.989 for final EE score (i.e., high or low) and also a high correlation for each EE component: initial statement (r = 0.96); warmth (r = 0.68); relationship (r = 0.84); positive comments (r = 0.81); and negative comments (r = 0.89). Percentage of transcription agreement was also high (27822/27892*100 = 99.7%).

Table 2 shows descriptive and between-group comparison data for Expressed Emotion (EE) and Activity questionnaire (WWP) data.

**Table 2:** Descriptive and between-group comparison data for Expressed Emotion (EE) and Activity questionnaire (WWP).

	Clinical group	Control group
**EE Total score**	Median = 5.76 (sd 1.3)*	Median = 8.62 (sd 0.67)*
**Initial Statement**	N- = 6, N = 10, P = 5 **	N- = 0, N = 1, P = 20**
**Relationship**	N- = 2, N = 17, P = 2 **	N- = 0, N = 6, P = 15 **
**Warmth**	N- = 6, N = 13, P = 2 **	N- = 0, N = 1, P = 20 **
**Positive comments**	M = 3.14 (sd 1.68)**	M = 9.76 (sd 3.72)**
**Negative comments**	M = 8.9 1 (sd 4.21)**	M = 2.00 (sd 2.21)**
**WWP mean score**	M = 41.71 (sd 8.574)**	M = 8.62 (sd 4.98)**

Mean values (M); Standard deviation (sd) in brackets; N- =  Negative comments; N =  Neutral comments; P =  positive comments; ** Denotes significant differences between groups (*p*≤0.001); * Denotes significant differences between groups (*p*≤0.01).

### Correlations between WWPS, EE and SES factors per group

Table 3 shows that among the control group, younger parents expressed more warmth, better relationships and more positive initial statements when talking about their children than older parents (r = −0.47, *p*<.05). Participants with older partners described their children as less active, obtaining lower scores in the WWP (r = 0.53, *p*<.05) than participants with younger partners. Number of siblings was also associated with lower or more positive EE scores as smaller number of siblings was associated with better parent-child relationships, warmth and initial statement (r = −0.54, *p*<.05). Age from participant and partner was associated with amount of income (r = 0.53 and r = 0.49 respectively, *p*<.05). And finally, a small number of siblings was associated with a higher level of participant education (r = −0.44, *p*<.05).

**Table 3:** Pearson correlations between Expressed Emotion (EE), Activity questionnaire (WWP^a^) and socio-demographic factors for control group.

Measure	2	3	4	5	6	7	8	9
**1. Total WWPS**	0.29	0.93	−0.17	0.01	−0.53*	0.05	−0.15	−0.34
**2. Total EE**	-	−0.14	0.02	−0.47*	−0.26	−0.54*	0.39	−0.08
**3. Negative comments**		-	−0.05	−0.02	0.01	0.02	0.01	0.05
**4. Positive comments**			-	−0.29	−0.34	0.16	−0.16	−0.31
**5. Age**				-	0.54*	0.26	−0.09	0.53*
**6. Partner**'**s age**					-	0.28	−0.09	0.49*
**7. Siblings**						-	−0.44*	−0.19
**8. Education**							-	0.18
**9. Income**								-

a Weiss Werry Peter Scale.

*p<0.05.

** p<0.001.

In the clinical group we found a strong correlation (r = 0.637, *p*<.001) between the EE components (warmth, a positive relationship and a positive initial statement about the pre-school child) and the number of positive comments. The results also indicated a strong positive correlation (r = 0.580, *p*<.05) between number of siblings and participant partner age (see Table 4).

**Table 4:** Correlations between WWPS, EE and socio-demographic factors for the clinical group.

Measure	2	3	4	5	6	7	8	9
**1. Total WWPS**	−0.29	0.40	−0.21	−0.21	−0.28	0.15	−0.27	−0.15
**2. Total EE**		−0.33	0.64*	0.88	0.91	0.26	0.37	0.74
**3. Negative comments**		-	0.16	−0.34	−0.19	0.21	0.28	−0.22
**4. Positive comments**			-	−0.31	−0.29	−0.28	0.08	0.08
**5. Age**				-	0.64*	0.31	−0.12	0.34
**6. Partner**'**s age**					-	0.58*	0.39	0.27
**7. Siblings**						-	0.23	0.28
**8. Education**							-	0.32
**9. Income**								-

*p<0.05.

** *p*<0.001.

### Corpus-based Linguistic Analysis

The keyword lists generated in Wordsmith Tools yielded 130 items for the control data, and 112 for the clinical data file. The items on the lists were then grouped together into semantic sets, comprising seven groups of words with related meanings. Table 5 shows the semantic categories identified, the number of different lexical items (types), and the number of actual instances of relevant lexis (tokens) identified within the keyword lists in each category. Full lists of keywords assigned to each category are provided in the analysis to follow.

**Table 5:** Semantic categories identified distinguishing between the number of different lexical items (types) per participant group, and the number of actual instances of relevant lexis (tokens) identified within the keyword lists in each category.

Semantic Category	Types		Tokens	
	Control	Clinical	Control	Clinical
**Affection**: *loves, caring, loving, cuddles…*	6	1	29	12
**Enjoyment/Positive Mood**: *likes, loves, enjoys…*	5	3	179	53
**Behaviour/Temperament/Attitude**: *inquisitive, cheeky, naughty…*	7	20	45	196
**Communication**: *interact, talks, listens…*	6	4	28	58
**Activities**: *playing, play, scooter, toys…*	15	2	110	51
**Routines**: *nursery, preschool, swimming…*	4	4	55	48
**People**: *brother, sister, friends…*	4	3	93	106

It must be stressed that the numbers of tokens or actual lexical items observed in the datasets relate only to those items that occur with a frequency that is sufficiently high to be recognized as keywords by the text analysis software (*p*≤0.00001). Thus, any lexical items with frequencies below the keyword threshold are not counted in the figures provided above. Of course, this is true of both datasets, a circumstance which to some extent must serve to attenuate any exaggeration of observed differences between the clinical and control data. However, it should nevertheless be noted that a comparison of the two datasets based on tokens counted only for those items which appear on keyword lists is necessarily selective.

With this caveat in mind, it is possible to make a number of observations from the results presented in Table 5. Of the seven semantic categories identified, only two, *routines* and *people*, exhibit no significant differences. Attention exclusively to the number of different keywords (types) assigned to each semantic category would suggest that the most marked differences between the clinical and control data pertain to lexis associated with *behaviour, temperament and attitude*, and also *activities*. However, when we consider the actual number of words spoken it becomes apparent that there exist significant differences between the two datasets for each of the five remaining semantic categories. These differences will be considered in more detail below.

### Affection

While the control data keyword analysis reveals seven different lexical items belonging to this semantic field, only one is evident in the clinical data (see Table 6).

**Table 6:** Lexical items within the category *Affection*.

Control	Clinical
caring (10)	loving (12)
cuddles (4)	
loving (6)	
affectionate (3)	
hugs (3)	
snuggle (3)	

Items from this semantic field other than *loving* are evident in the clinical data, but only *loving* occurs with a frequency that is statistically significant. This item in fact occurs twice as frequently in the clinical data than in the control data, but there is evidence of qualification in the clinical data that is absent from the control data. In the clinical dataset, *loving* is followed on 4 occasions by lexis referring to negatively evaluative attributes, such as *handful, clingy, bad tempered* and *hyper*. It is also pre-modified by *overly* on one occasion, and on another features as complement in the clause *he can be very loving*. This latter construction occurs some 19 times in the clinical data, where it serves to emphasize capability rather than habitual behaviour.

### Enjoyment and positive mood

The most salient item in this category is *likes*, which occurs 105 times in the control data and 38 times in the clinical data (see Table 7).

**Table 7:** Lexical items within category *Enjoyment/Positive Mood*.

Control	Clinical
likes (105)	likes (38)
happy (20)	loves (12)
loves (31)	enjoys (5)
enjoys (10)	
fun (13)	

Of course, insofar as that it is possible to *like* generally undesirable phenomena, this item can be used in a negatively evaluative way, a usage which is apparent in both datasets. But while *like* appears to be used in a negative context only four times in the control data, it is used in this way nine times in the clinical data, in utterances such as *likes to climb on the table, likes to interrupt*, and *likes to pinch*.

### Behaviour, temperament and attitude

Some overlap between the two datasets is clearly evident with regard to lexis relating to *behaviour, temperament and attitude*, specifically the items *naughty* and *tantrum* which appear in the keyword lists for both datasets (see Table 8). Indeed, *tantrum* features with comparable frequency in the clinical and control data, but this seems to be where the similarities end. The most immediately obvious difference between the two keyword lists with regard to this semantic field relates to the number of *types* or different words; the clinical data features some 20 types compared to just seven for the control data. It is also immediately apparent that while all but two of the control group items are positively evaluative, most of those found in the clinical data appear to be negatively evaluative.

**Table 8:** Lexical items within category *Behaviour/Temperament/Attitude*.

Control	Clinical
inquisitive (7)	naughty (19)
cheeky (9)	constantly (15)
lively (7)	frustrated (12)
naughty (9)	difficult (25)
excited (6)	behaviour (13)
tantrum (4)	sit (25)
sociable (3)	hyper (6)
	struggles (5)
	tantrum (5)
	hyperactive (4)
	handful (6)
	hitting (7)
	challenging (6)
	handful (6)
	boisterous (4)
	throw (11)
	mardy (3)
	running (about/around/off) (15)
	screaming (5)
	temper (4)

The item *difficult* merits further explanation, since of the 25 instances of this item in the clinical data, only six are predicated upon a human agent, as in *X can be very difficult* or *he*'*s quite difficult*. The remaining 13 instances of *difficult* describe situations or circumstances rather than individuals. However, in each of these instances the situations or circumstances described relate, not surprisingly given the topic of discussion, directly to dealing with the child in question. This can be observed in utterances such as *I do find it difficult to communicate with him*, and *X finds it quite difficult to listen sometimes and struggles to gain eye contact*. Thus, even where *difficult* does not describe a quality attributed to the child being discussed, it nevertheless refers to circumstances that relate to the child in some way.

### Communication

While the number of *types* relating to communication is slightly greater for the control data, the actual numbers of items (tokens) is far greater for the clinical dataset, with 79 items in all compared to 40 for the control data (see Table 9).

**Table 9:** Lexical items within category *Communication*.

Control	Clinical
interact (4)	listen (18)
attentive (3)	understand (21)
eloquent (3)	communicate (6)
talks (7)	speech (13)
interaction (4)	
conversations (7)	

However, while all of these items in the control group data are framed positively, they are more often negated or otherwise problematized in the clinical data. For example, of the 18 instances of *listen* in the clinical data, 11 are directly negated, two framed in terms of difficulty as in *it*'*s difficult for her to listen* and *X finds it difficult to listen*, while all are problematized in some way. Similarly, *understand* is negated in 16 out of 21 instances, while *communicate* is preceded by *difficult* three times and *hard* once, and is problematized in all six occurrences. This suggests that parent-child communication is a more salient topic for the participants in the clinical group, and that the reason for this salience is its problematic nature for these parents.

### Activities

While the control group keyword list features 15 items relating to activities, only two such items appear on the keyword list for the clinical dataset (see Table 10).

**Table 10:** Lexical items within category *Activities*.

Control	Clinical
play (29)	play (36)
playing (30)	toys (15)
scooter (7)	
park (16)	
stories (11)	
swimming (11)	
games (10)	
trampoline (4)	
puzzles (4)	
bedtime (4)	
colouring (5)	
climbing (5)	
watch (15)	
learning (8)	
pirates (3)	
drawing (7)	

In fact, a number of other, *non-key* items relating to play and activities can be found in the clinical data, where for example *park* and *telly* each occur six times, *reading* twice and also *playing* 16 times. However, the broader range of items evident in the control data, and the fact that 15 of these items occur with a frequency that is statistically significant compared to only two in the clinical data, provides strong evidence to suggest that talk relating to play and activities is a less salient category in the clinical data than might be expected. The most frequently occurring item in this category for the clinical data is *play*, an item which actually occurs with greater frequency in the clinical than control data at 36 occurrences compared to 29. However, closer analysis reveals important differences in the use of this item in the two datasets. Of the 36 instances of *play* in the clinical data, 16 are either negated or marked as problematic in some sense. These items are presented in context in the concordance output in Appendix S1. No equivalent pattern is observed in the control data, where all instances of *play* are positively evaluative. Of particular interest are lines 2, 3, 5, 8, 11, 12 and 14, all of which problematize an unwillingness or inability to play alone. It is also interesting to consider the item *attention* in the two datasets, an item which appears on the keyword list for clinical data with 35 occurrences, but not on that generated for the control data, in which it occurs just five times. Of the 35 instances of this item in the clinical data, 18 unambiguously relate to a need for parental attention that is framed as excessive.

### Mitigating syntax

A prominent pattern observed in both datasets is ‘*x* can be *y*’, where *x* = a proper name or personal pronoun and *y* = an attribute or behaviour (see Table 11). This modal construction is interesting because it places an emphasis on capability or possibility, in this way foregrounding or emphasizing the transitory nature of the behaviour described. Evidence for this interpretation can be found in the 10 million word spoken component of the British National Corpus [66], where the most significant collocate of *can be + adjective* is *sometimes*, an adverb typically used to foreground the occasional nature of events or behaviours.

**Table 11:** Mitigating syntax items ‘*x* can be *y*’.

Control	Clinical
a bit mischievous	a bit challenging
a cheeky monkey	a handful
quite awkward	quite boisterous
quite difficult	really spiteful
trying sometimes	very aggressive
	very challenging
	very difficult (x2)

The *x can be y* pattern is more frequent in the clinical data with 19 instances than in the control data with just 12 instances. There are also some interesting differences in the ways in which this phrasing is used in the two datasets (see Appendix S2 for full concordance outputs). The qualities or behaviours referred to in this phrase can be roughly separated into instances that are positively or negatively evaluative, and also a small number which are ambiguous in this regard.

Examination of the positively evaluative instances of this phrase is also revealing. In both datasets positive evaluation is contextualized alongside other behaviours which are negatively evaluated, and indeed this seems to be one of the principle functions of this particular phraseology in the data presented here (see Table 12).

**Table 12:** Mitigating syntax used for positive instances.

Control	Clinical
very kind and caring (to his sister, but equally he can push her over)	a good lad (can't he sometimes)
	a really good child
very kind (equally he can be a cheeky monkey)	happy (one minute and flip the next)
	happy playing (he can go into a right mood)
very thoughtful (equally he can be a cheeky monkey)	lovely at times
very thoughtful	really good (she has really nice moments where she can be)
	so nice (one minute)
	very loving (when you get him to sit down for 5 minutes)
	very loving (on the flip side)

However, there appears to be more of a focus on the temporary nature of positively evaluated behaviour in the clinical group; he can be a good lad *sometimes*, she can be good… she can have *really nice moments*, he can be very loving … *when you get him to sit him down for five minutes*.

## Discussion

Analysing *what* parents say about their children and *how* they say it extends and innovates on prior ADHD work by directly comparing and contrasting linguistic data from parents of children at risk of developing ADHD with controls to further understand parents' views and maximise the relevancy of parenting interventions. The general pattern that emerges in the corpus based linguistic analysis can be described in terms of a clear tendency towards negative evaluation in the clinical data. Evidence of positively evaluative language is evident, but this is frequently accompanied by mitigating lexis or syntactic structures. An example of the latter was observed in the modal construction *x can be y*, which was seen to limit the extent of any positive evaluation that might obtain when referring to actions that might normally be subject to such evaluation. The semantic categorisation of keywords made it possible to pin-point specific spheres of activity that are represented by the clinical group as problematic. Significant differences between the two datasets were observed in five of the seven semantic categories identified, suggesting marked differences in the preoccupations and evaluative representations evident in the clinical and control groups. The item *attention* in the two datasets, an item which appears on the keyword list for clinical data with 35 versus five occurrences in the control data provides strong evidence to suggest a general concern with the demands for attention made by the children discussed in the clinical group, while play seems to be an activity in which such demands are particularly salient for these parents.

Regarding the pattern *x can be y*, findings demonstrate that parents from the clinical group produce this mitigating syntax more frequently in negatively evaluative instances than controls. When we compare the negatively evaluative instances of this pattern in the two datasets interesting differences become evident. In both data sets we see evidence of attenuation – in the clinical data a *bit* challenging, *quite* boisterous, *quite* feisty (although it should be noted that *quite* can function as an intensifier rather than an attenuator). In the control data we see similar patterns – *a bit* mischievous, *a bit* wingey, *quite* awkward, *quite* difficult, trying *sometimes*. However, in the clinical data intensification is also in evidence – *really* spiteful, *very* aggressive, *very* challenging, *very* difficult, while the actual qualities attributed to the subject of the clause– *spiteful, aggressive* and *difficult* compared to *a cheeky monkey, wingey* and *awkward*, are arguably more extreme. When we compare the positively evaluative instances of this pattern, we observed that while negative behaviour is more negative in the clinical data, positive behaviour, when it is referred to, is often framed as a temporary aberration from a more negatively evaluated norm.

Parenting interventions may build upon these linguistic differences to ensure parents' unique experiences, preferences, and goals are met. Our results identify behavioural and emotional aspects important to guide interventions. We will explore the practical implications derived from the five semantic categories exhibiting significant differences between groups.

### Affection

Parents from the clinical sample described their children as less affectionate than controls. ADHD symptoms and associated negative and incompliant behaviours [69], [70] can lead to parental challenges [71], parental anxiety, and mood problems [72]. On the other hand, parental depression is one of the most well-established risk factors for adverse child development and psychopathology [73] and occurs more often in parents of children with ADHD [74]. Parents with depression engage in more threatening, hostile, and coercive behaviour, are more disengaged and withdrawn during interactions with their child, and are less involved in positive parenting behaviours including displays of praise and affectionate contact [75]. If parental wellbeing has a transactional relationship with difficult behaviour [76], parenting interventions should highlight the importance of praise, positive tone of voice, mutual respect, and physical affection as well as techniques to increase parental wellbeing.

### Enjoyment and positive mood

Parents from the clinical sample described their children as enjoying less and having less fun than controls. Previous research has shown that children with ADHD have a different sensitivity to reward [77]. They prefer smaller, immediate rewards over larger later rewards [78] and display greater sensitivity to social rewards [79]. Parenting interventions should be aware of these differences and promote games in which rewards are immediate and frequent, ensuring children engage and participate in playing and fun activities. Behaviour should be reinforced as frequently and immediately as possible and whenever appropriate use ‘ear shot’ praising techniques. For example, when the child can hear Mum/Dad conversing with another person, Mum/Dad mentions how proud she/he is because the child was affectionate or playing gently with a smaller cousin. The importance of play has already been highlighted by existing parenting interventions [80]. Play supports child development and it can improve the child attention, concentration and listening skills, but it is also a great opportunity to have fun and enjoy parenting.

### Behaviour, temperament and attitude

Parents from the clinical sample described their children as displaying more negative behaviour, temperament and attitude than controls. Effective parenting interventions should emphasise strategies for dealing with temper tantrums and disruptive behaviour. Research has shown that ADHD children may exhibit deficits in temporal processing [81], time discrimination, and reproduction [82] which may explain difficulties in awaiting turns and interruptions. Temporal processing deficits ameliorate with reinforcement in children with ADHD [83]. It may, therefore, be advantageous for parents to routinely set clear instructions about how long a child needs to engage in behaviours in order to receive a reward and use timers. The New Forest Parenting Training (NFPP) [84], [85] highlights the importance of practising time management using buzzers, timers, and alarms, and also the use of countdowns and warnings (e.g., ‘we are leaving in ten minutes, we are leaving in five minutes, and so on’ vs. ‘Get your jacket, it’s time to go’). For children with ADHD, ‘quiet time’ has been suggested to be more effective then ‘time out’. While ‘quiet time’ promotes reflection, negotiation, self-regulation, and is not perceived as a punishment, ‘time out’ is a more extreme measure and should only be used as a last resort when the child's behaviour is unacceptable but distraction, quiet time, presenting the child with choices and other strategies have not worked [85].

### Communication

Parents from the clinical sample described their children as displaying more communication problems than controls. Parenting interventions can help both parents and children with their communication skills. For example, the parenting programme ‘1-2-3 Magic’ [86] pays special attention to sympathetic listening (e.g., reflective feelings), while NFPP focuses on more basic communication skills such as active listening (e.g., repeating the message using the same or similar words), making eye contact, and getting the child's attention before giving any instructions. To expand the child language through play, NFPP recommends using descriptive comments and explanations to keep the child interested in carrying on playing while increasing his vocabulary. NFPP also focuses on communication aspects such as voice (e.g., volume and tone), for example, voice should be calm in difficult situations, firm when giving instructions and positive when rewarding and reinforcing behaviour. Parents are also encouraged to give children just two choices to help them decide and also to remember their choice, avoid confrontations, and arguments. The NFPP pays special attention to setting clear goals (e.g., short and simple sentences) and scaffolding which consists on identifying what the child is able to do, extending these abilities by supporting the child in tasks that present moderate challenges and practising them to ensure effective learning. Moreover, positive communication including low levels of criticism and hostility and high levels of warmth and empathy have been shown positively influence the pathogenic mechanism of ADHD [26].

### Activities

Parents from the clinical sample described playing time as problematic and also reported less variety of activities. The Incredible Years parenting programme [87] pays special attention to self-directed play to promote positive relationships and encourage parents to pay attention and play with their children for at least 10 minutes every day. ‘1-2-3- Magic’ encourages parents to share one-to-one fun activities with their child to promote bonding. The NFPP also provides support for managing the child outside of the home promoting outdoor activities and skills that facilitate ‘teachable moments’, for example, asking the child in the supermarket to collect two tins of tomatoes that look the same. Weekly income could affect how much money a parent can invest in their child and therefore it could limit the activities they do together [88]. Even though most of the activities evident only in the control group are inexpensive (e.g., colouring, drawing, and going to the park) and income did not affect EE and WWP ratings, we cannot exclude that economic factors could explain some of the differences observed in the activities reported by both groups.

### Mitigating syntax

Parents from the clinical sample evaluated their children more negatively than controls and when they expressed a positive evaluation mitigated it by adding a temporal factor. As discussed above, praise and ‘ear shot’ techniques are especially relevant for children exhibiting early ADHD symptoms as they are especially sensitive to social reward [85]. Reinforcement is a key component of most parenting interventions and may help to establish target behaviours win children with ADHD, especially those with abnormalities in reward processing. For example, functional Magnetic Resonance Imaging (fMRI) data have shown that reinforcement has a comparable effect on brain function in children with ADHD as medication [89].

## Conclusions and Recommendations

The general pattern that emerges in the corpus based linguistic analysis can be described in terms of a clear tendency towards negative evaluation in the clinical data. Evidence of positively evaluative language is evident, but this is frequently accompanied by mitigating lexis or syntactic structures. An example of the latter was observed in the modal construction *x can be y*, which was seen to limit the extent of any positive evaluation that might obtained when referring to actions that might normally be subject to such evaluation. The semantic categorisation of keywords made it possible to pin-point specific spheres of activity that are represented by the control group parents as problematic. Significant differences between the two datasets were observed in five of the seven semantic categories identified, suggesting marked differences in the preoccupations and evaluative representations evident among parents of preschool children with the hyperactive/impulsive subtype of ADHD and control groups.

The findings reported in this article show that corpus-based linguistic analysis is a useful and effective method to further understand parents' unique perceptions of children with ADHD symptoms. Corpus-based linguistic analysis offers new understandings and insights about topics that otherwise may be uncovered. These new insights might help researchers, educators and healthcare professionals to improve the parent-child relationship by strengthening the relevancy and therefore success and acceptability of parenting interventions.

The parents' narratives analysed in this study provide evidence on how parenting programmes should include not just techniques to improve effective behaviour management and communication skills but also strategies that focus on promoting affectionate parent-child relationships, positive perceptions, and activities that facilitate enjoyment and positive mood within the family context. General praise, specific ‘ear-shot’ praising techniques, positive tone of voice, mutual respect, physical affection, tailored games with immediate and frequent reward, planning activities, and dedicated daily play time are essential aspects that parenting interventions for children with early signs of ADHD should address and weight accordingly.

A future follow-up study could identify those parents from the clinical group that have participated in a parenting programme and compare their post-intervention FMSS with the one collected for the current study (i.e., baseline). These two FMSS measures would allow us to further assess parenting intervention outcomes. Because the way parents describe their children can moderate the relationship between the genetic risk for ADHD and the expression of ADHD symptoms [26], future research should also focus on designing behavioural interventions able to reduce negative Expressed Emotion.

Further studies focusing on family influences and family characteristics are required to establish the direction of causation and extend our understanding of the relationship between Parental Expressed Emotion, ADHD and parenting programmes. A recent meta-analysis on behavioural interventions in ADHD [90] has demonstrated that the value of these interventions does not rest exclusively on the potential effects on child ADHD symptoms but on associated features including child conduct problems, academic performance and social skills. Interestingly, outcomes on parenting functioning and parenting sense of competence were also improved, indicating than when parents successfully engage in behavioural interventions that focus on making parenting more enjoyable, implementing new strategies to manage challenging behaviour, consistency and family routine, those parents report not only an increment on positive parenting but a significant improvement on self-efficacy and self-concept. Behavioural interventions, however, seem to have a limited effect on parental mental health highlighting that the high levels of mental health problems often reported among parents of children with ADHD are not solely the result of issues around parenting, but rather reflect a shared genetic risk for mental health problems within families.

## Limitations

Administration of the PFMSS and demographics questionnaire differed between groups. While both measures for the clinical group were collected face-to-face at the parents' home, these measures for the control group were collected over the phone. The WWS was collected over the phone for both groups. Face-to-face and telephone communication does differ, however, research on FMSS collection has shown that this does not affect results [91]. Educational level was an unmatched demographic characteristic that influenced the EE scorings. Interestingly, income could not be matched but did not affect any of the scorings.

Another limitation of this study is the small sample size and the fact that ADHD symptomatology was not assessed in the participating parents.

## Supporting Information

Appendix S1 (DOCX)

Appendix S2 (DOCX)

## References

[1] Polanczyk G, Rohde LA (2007) Epidemiology of attention-deficit/hyperactivity disorder across the lifespan. Current Opinion Psychiatry, 20: ,386–392. 10.1097/YCO.0b013e3281568d7a 17551354

[2] National Institute for Health and Clinical Excellence (2008) Clinical guideline 672: Attention Deficit Hyperactivity Disorder (ADHD) Full Guideline, London: National Institute of Clinical Excellence. Available: www.nice.org.uk/CG72.

[3] Loe IM, Feldman HM (2007) Academic and educational outcomes of children with ADHD. Ambulatory Paediatrics 7: 82–90. 10.1016/j.ambp.2006.05.005 17261487

[4] Swanson JM, Sergeant JA, Taylor E, Sonuga-Barke EJS, Jensen PS, et al (1998) Attention-deficit hyperactivity disorder and hyperkinetic disorder. Lancet 351: 429–433. 9482319

[5] Biederman J, Monuteaux MC, Mick E, Spencer T, Wilens TE, et al (2006) Psychological Medicine. 36: 167–179. 10.1017/S0033291705006410 16420713

[6] Klassen AF, Miller A, Fine S (2004) Health-related quality of life in children and adolescents who have a diagnosis of Attention-Deficit/Hyperactivity Disorder. Paediatrics 114: e541–547. 10.1542/peds.2004-0844 15520087

[7] Pelham W, Foster E, Robb J (2007) The economic impact of attention-deficit/hyperactivity disorder in children and adolescent. Journal of Pediatrics Psychology 32 711–727. 10.1093/jpepsy/jsm022 17556402

[8] Taylor E, Dopfner M, Sergeant J, Asherson P, Banaschewski T, et al (2004) European clinical guidelines for hyperkinetic disorder: first upgrade. European Child and Adolescent Psychiatry 15: 476–495. 10.1007/s00787-004-1002-x 15322953

[9] Banaschewski T, Coghill D, Santosh P, Zuddas A, Asherson P, et al (2006) Long-acting medications for the hyperkinetic disorders: a systematic review and European treatment guideline. European Child and Adolescent Psychiatry 15: 476–495. 16680409 10.1007/s00787-006-0549-0

[10] Sonuga-Barke E, Brandeis D, Cortese S, Daley D, Ferrin M, et al (2013) Nonpharmacological Interventions for ADHD: Systematic Review and Meta-Analyses of Randomized Controlled Trials of Dietary and Psychological Treatments. American Journal of Psychiatry 170(3): 275–289. 23360949 10.1176/appi.ajp.2012.12070991

[11] van de Loo-Neus GH, Rommelse N, Buitelaar JK (2011) To stop or not to stop? How long should medication treatment of attention-deficit hyperactivity disorder be extended? European Neuropsychopharmacology 21: 584–599. 21530185 10.1016/j.euroneuro.2011.03.008

[12] Graham J, Banaschewski T, Buitelaar J, Coghill D, Danckaerts M, et al (2011) European Guidelines Group: European Guidelines on managing adverse effects of medication for ADHD. European Child and Adolescent Psychiatry 20: 17–37. 21042924 10.1007/s00787-010-0140-6 PMC3012210

[13] Berger I, Dor T, Nevo Y, Goldzweig G (2008) Attitudes toward attention-deficit hyperactivity disorder (ADHD) treatment: parents' and children's perspectives. Journal of Child Neurology 23: 1036–1042. 18487521 10.1177/0883073808317726

[14] Nigg JT, Lewis K, Edinger T, Falk M (2012) Meta-analysis of attention-deficit/hyperactivity disorder or attention-deficit/hyperactivity disorder symptoms, restriction diet, and synthetic food color additives. Journal of the American Academy of Child and Adolescent Psychiatry 51: 86–97. 22176942 10.1016/j.jaac.2011.10.015 PMC4321798

[15] Bloch MH, Qawasmi A (2011) Omega-3 fatty acid supplementation for the treatment of children with attention-deficit/hyperactivity disorder symptomatology: systematic review and meta-analysis. Journal of the American Academy of Child and Adolescent Psychiatry 50: 991–1000. 21961774 10.1016/j.jaac.2011.06.008 PMC3625948

[16] Markomichali P, Donnell N, Sonuga-Barke E (2009) Cognitive training for attention, inhibition and working memory deficits: a potential treatment for ADHD? Advances in ADHD 3: 89–96.

[17] Arns M, de Ridder S, Strehl U, Breteler M, Coenen A (2009) Efficacy of neurofeedback treatment in ADHD: the effects on innatention, impulsivity, and hyperactivity: a meta-analysis. Clinical Psychology Review 40: 180–189. 10.1177/155005940904000311 19715181

[18] Fabiano GA, Pelham WE Jr, Coles EK, Gnagy EM, Chronis-Tuscano A, et al (2009) A meta-analysis of behavioural treatments for attention-deficit/hyperactivity disorder. Clin Psyhcol Rev 29: 180–189. 10.1016/j.cpr.2008.11.001 19131150

[19] Bor W, Sanders M, Markie-Dadds C (2002) The effects of the Triple P-Positive Parenting Programme on preschool children with co-occurring disruptive behavior and attentional/hyperactive difficulties. J Abnorm Child Psycho 30: 571–587. 10.1023/a:1020807613155 12481972

[20] Thompson MJ, Laver-Bradbury C, Ayres M, Le Poidevin E, Mead S, et al (2009) A small-scale randomized controlled trial of the revised New Forest Parenting Package for preschool children with Attention Deficit Hyperactivity Disorder. Eur Child Adolesc Psychiatry 18: 605–616. 19404717 10.1007/s00787-009-0020-0

[21] Brown R, Khan L, Parsonage M (2012) A Chance to Change. Delivering effective parenting programmes to transform lives. Annual Report Centre for Mental Health. London.

[22] Deault L (2010) A systematic review of parenting in relation to the development of comorbidities and functional impairments in children with attention-deficit/hyperactivity disorder (ADHD). Child Psychiatry Human Development 41(2): 168–192. 19768532 10.1007/s10578-009-0159-4

[23] Johnston C, Mash EJ (2001) Families of children with attention-deficit/hyperactivity disorder: review and recommendations for future research. Clinical Child and Family Psychology Review 4(3): 183–207. 11783738 10.1023/a:1017592030434

[24] Thapar A, Cooper M, Jefferies R, Stergiakouli E (2012) What causes attention deficit hyperactivity disorder? Archives of disease in childhood 97(3): 260–265. 21903599 10.1136/archdischild-2011-300482 PMC3927422

[25] Rutter M, Moffitt TE, Caspi A (2006) Gene-environment interplay and psychopathology: multiple varieties but real effects. J Child Psychol Psychiatry 47: 226–261. 16492258 10.1111/j.1469-7610.2005.01557.x

[26] Sonuga-Barke S, Lasky-Su J, Neale B, Oades R, Chen W, et al. (2008). Does parental expressed emotion moderate genetic effects in ADHD? An exploration using a genome wide association scan. Am J Med Gen 147B: , 1359–1368. 10.1002/ajmg.b.30860 18846501

[27] Hughes C (2011) Changes and challenges in 20 years of research into the development of executive functions. Infant and Child Development 20(3): 251–271.

[28] Sonuga-Barke E, Thompson M, Abikoff H, Klein R, Brotman LM (2006) Nonpharmacological interventions for preschoolers with ADHD: the case for specialized parent training. Infants & Young Children 19(2): 142–153.

[29] Ainsworth M (1979) Infant-mother attachment. American Psychologist 34: 932–937. 517843 10.1037//0003-066x.34.10.932

[30] Ainsworth M (1989) Attachments beyond infancy. American Psychologist 44: 709–716. 2729745 10.1037//0003-066x.44.4.709

[31] Shaver P, Mikulincer M (2010) New directions in attachment theory and research. Journal of Social and Personal Relationships 27: 163–172.

[32] Simpson J, Rholes W (2010) Attachment and relationships: Milestones and future directions. Journal of Social and Personal Relationships 27: 173–180.

[33] Lench H, Levine L, Whalen C (2011) Exasperating or Exceptional? Parent's Interpretations of Their Child's ADHD Behaviour. Journal of Attention Disorders 17(2): 141–151. 22166469 10.1177/1087054711427401

[34] Fiks A, Gafen A, Hughes C, Hunter K, Barg F (2011) Using freelisting to understand shared decision making in ADHD: Parent's and pediatricians' perspectives. Patient Education Counseling 84: 236–244. 20797833 10.1016/j.pec.2010.07.035 PMC3551534

[35] Haidet P, O'Malley KJ, Sharf BF, Gladney AP, Greisinger AJ, et al (2008) Characterizing explanatory models of illness in healthcare: development and validation of the CONNECT instrument. Patient Education Counseling 73: 232–239. 18760889 10.1016/j.pec.2008.07.007

[36] Bussing R, Koro-Ljungberg M, Williamson P, Garye F, Wilson C (2006) What “Dr. Mom” ordered: A community-based exploratory study of parental self-care responses to children's ADHD symptoms. Social Science & Medicine 63(4): 871–882. 16644078 10.1016/j.socscimed.2006.03.014

[37] Hermansen M, Miller P (2008) The lived experiences of mothers of ADHD children undergoing chiropractic care: A qualitative study. Clinical Chiropractic 11(4): 182–192.

[38] Magana A, Goldstein M, Karno M, Miklowitz D, Jenkins J, et al (1986) A brief method for assessing expressed emotion in relatives of psychiatric patients. Psychiatry Research 17: 203–212. 3704028 10.1016/0165-1781(86)90049-1

[39] Hooley J (1985) Expressed emotion: a review of the critical literature. Clinical Psychology Review 5: 119–139.

[40] Baker B, Heller T, Henker B (2000) Expressed Emotion, Parenting Stress, and Adjustment in Mothers of Young Children with Behavior Problems. Journal of Child Psychology and Psychiatry 41(7): 907–915. 11079433

[41] Cartwright KL, Bitsakou P, Daley D, Gramzow RH, Psychogiou L, et al (2011) Disentangling Child and Family Influences on Maternal Expressed Emotion Toward Children With Attention-Deficit/Hyperactivity Disorder. Journal of the American Academy of Child and Adolescent Psychiatry 50(10): 1042–1053. 21961778 10.1016/j.jaac.2011.07.006

[42] Stubbe DE, Zahner GEP, Goldstein MJ, Leckman JF (1993) Diagnostic specificity of a brief measure of expressed emotion: a community study of children. Journal of Child Psychology and Psychiatry 34: 139–154. 8444989 10.1111/j.1469-7610.1993.tb00976.x

[43] Rogosch FA, Cicchetti D, Toth SL (2004) Expressed emotion in multiple subsystems of the families of toddlers with depressed mothers. Development and Psychopathology 6(3): 689–709. 10.1017/s0954579404004730 15605632

[44] Psychogiou L, Daley D, Thompson M, Sonuga-Barke E (2007) Mother's expressed emotion toward their school-aged sons. Associations with child and maternal symptoms of psychopathology. Eur Child Adolesc Psychiatry 16: 458–464. 17876512 10.1007/s00787-007-0619-y

[45] Cussen A, Sciberras E, Ukoumunne O, Efron D (2012) Relationship between symptoms of attention-deficit/hyperactivity disorder and family functioning: a community-based study. Eur J Pediatr 171: 271–280. 21743986 10.1007/s00431-011-1524-4

[46] Keown L (2010) Fathering and mothering of pre-school boys with hyperactivity. Int J Behav Dev 35: 161–168.

[47] Cartwright K, Bitsakou P, Daley D, Gramzow R, Psychogiou L, et al (2011) Disentangling child and family influences on maternal expressed emotion toward children with attention-deficit/hyperactivity disorder. J Am Acad Child Adolesc Psychiatry 50: 1042–1053. 21961778 10.1016/j.jaac.2011.07.006

[48] Sonuga-Barke S, Cartwright K, Tompson M, Brow J, Bitsakou P, et al (2013) Family Characteristics, Expressed Emotion, and ADHD. J Am Acad Child Adolesc Psychiatry 50: 547–548. 10.1016/j.jaac.2013.03.005 23622856

[49] Richards J, Vasquez A, Rommelse N, Oosterlaan J, Hoekstra P, et al (2014) A Follow-up study on maternal expressed emotion toward children with attention-deficit/hyperactivity disorder (ADHD): Relation with severity and Persistence of ADHD and Comorbidity. J Am Acad Child Adolesc Psychiatry 53: 311–319. 24565358 10.1016/j.jaac.2013.11.011 PMC4066112

[50] Daley D, Sonuga Barker E, Thompson M (2003) Assessing expressed emotion in mothers of preschool AD/HD children: psychometric properties of a modified speech sample. British Journal of Clinical Psychology 42: 53–67. 12675979 10.1348/014466503762842011

[51] Wearden AJ, Tarrier N, Barrowclough C (2000) A review of expressed emotion research in health care. Clinical Psychology Review 20: 633–666. 10860170 10.1016/s0272-7358(99)00008-2

[52] Routh D (1978) Hyperactivity. In: Magrab P (Ed) Psychological Management of Paediatric Problems. 2^nd^ edition. Baltimore: University Park Press.

[53] Thompson M, Stevenson J, Sonuga-Barke, Nott E, Bhatti Z, et al (1996) The mental health of preschool children and their mothers in a mixed urban/rural population: I. Prevalence and ecological factors. British Journal of Psychiatry 168(1): 16–20. 8770422 10.1192/bjp.168.1.16

[54] Barkley RA (1988) Attention deficit disorder with hyperactivity. In Mash EJ, Terdal LJ (Eds) Behavioral assessment of childhood disorders (pp. 69: –104) New York: Guilford.

[55] Mash EJ, Johnson C (1983) Parental perceptions of child behaviour problems, parenting self-esteem, and mothers' reported stress in younger and older hyperactive and normal children. Journal of Consulting and Clinical Psychology 51: 86–99. 6826870 10.1037//0022-006x.51.1.86

[56] Sonuga-Barke E, Stevenson J, Thompson M, Viney D (1997) The structure of British pre-school and children's behaviour problems. Psychological Medicine 27: 909–918. 9234468 10.1017/s0033291797005291

[57] Cohen J (1988) Statistical Power Analysis for the Behavioural Sciences (2^nd^ Ed) LEA Publishers. Hillsdale, New Jersey.

[58] Partington A, Morley J, Haarman L, Eds. (2004) Corpora and Discourse. Bern: Peter Lang.

[59] Crawford P, Brown B, Nerlich B, Koteyko N (2008) The moral careers of microbes and the rise of the matrons: An analysis of UK national press coverage of methicillin resistant staphylococcus aureus (MRSA) 1995–2006. Health, Risk & Society 10(4): 331–347.

[60] Crawford P, Gilbert P, Gilbert J, Gale C, Harvey K (2013) The Language of Compassion in Acute Mental Health Care. Qualitative Health Research 23(6): , 719–727. 10.1177/1049732313482190 23515298

[61] Adolphs S, Crawford P, Brown B, Sahota O, Carter RA (2004) Applying Corpus Linguistic in a health care context. International Journal of Applied Linguistics, 1(1): , 44–49.

[62] Brown B, Crawford P (2009) Post antibiotic apocalypse: Discourses of mutation in narratives of MRSA. Sociology of Health & Illness 31(4): 508–524. 19144082 10.1111/j.1467-9566.2008.01147.x

[63] Harvey K, Churchill D, Crawford P, Brown B, Mullany L, et al (2008) Health communication and adolescents: What do their emails tell us? Family Practice 25: 1–8. 18562334 10.1093/fampra/cmn029

[64] Scott M (2012) WordSmith Tools. Version 6, Liverpool: Lexical Analysis Software.

[65] Baker P (2006) Using Corpora in Discourse Analysis. London: Continuum.

[66] British National Corpus (BNC XML Edition v3) Distributed by Oxford University Computing Services on behalf of the BNC Consortium. Available: http://www.natcorp.ox.ac.uk/

[67] Bradley HCR (2002) Socioeconomic status and child development. Annual Review of Psychology 53: 371–399. 10.1146/annurev.psych.53.100901.135233 11752490

[68] Ermisch JPC (2010) Causal Effects of Parents' Education on Children's education. Institute for Social and Economic Research.

[69] Johnston C (1996) Parent characteristics and parent-child interactions in families of non-problem children and ADHD children with higher and lower levels of oppositional-defiant behaviour. Journal of Abnormal Child Psychology 24: 85–104. 8833030 10.1007/BF01448375

[70] Johnston C, Murray C, Hinshaw SP, Pelham WE, Hoza B (2002) Responsiveness in interactions of mothers and sons with ADHD: Relations to maternal and child characteristics. Journal of abnormal child psychology 30(1): 77–88. 11930974 10.1023/a:1014235200174

[71] Pelham W, Lang A (1999) Can your children drive you to drink? Stress and parenting in adults interacting with children with ADHD. Alcohol Research and Health 23: 292–298. 10890826 PMC6760385

[72] Gerdes A, Hoza B, Arnold L, Pelham W, Swanson J, et al (2007) Maternal depressive symptomatology and parenting behaviour: Exploration of possible mediators. Journal of Abnormal Child Psychology 35: 705–714. 17674187 10.1007/s10802-007-9134-3

[73] Goodman SH, Gotlib IH (1999) Risk for psychopathology in the children of depressed mothers: a developmental model for understanding mechanisms of transmission. Psychological Review 106(3): 458. 10467895 10.1037/0033-295x.106.3.458

[74] Chronis AM, Lahey BB, Pelham WE, Kipp HL, Baumann BL, et al (2003) Psychopathology and substance abuse in parents of young children with attention-deficit/hyperactivity disorder. Journal of the American Academy of Child & Adolescent Psychiatry 42: 1424–1432. 14627877 10.1097/00004583-200312000-00009

[75] Lovejoy MC, Graczyk PA, O'Hare E, Neuman G (2000) Maternal depression and parenting behavior: A meta-analytic review. Clinical Psychology Review 20(5): 561–592. 10860167 10.1016/s0272-7358(98)00100-7

[76] Goodman SH, Rouse MH, Connell AM, Broth MR, Hall CM, et al (2011) Maternal depression and child psychopathology: a meta-analytic review. Clinical Child and Family Psychology Review 14(1): 1–27. 21052833 10.1007/s10567-010-0080-1

[77] Sonuga-Barke E (2011) Editorial: ADHD as a reinforcement disorder: moving from general effects to identifying (six) specific models to test. Journal of Child Psychology and Psychiatry 52(9): 917–918. 21793824 10.1111/j.1469-7610.2011.02444.x

[78] Sonuga-Barke E, Taylor E, Sembi S, Smith J (1992) Hyperactivity and Delay Aversion—I. The Effect of Delay on Choice. Journal of Child Psychology and Psychiatry 33(2): 387–398. 1564081 10.1111/j.1469-7610.1992.tb00874.x

[79] Kohls G, Peltzer J, Herpertz-Dahlmann B, Konrad K (2009) Differential effects of social and non-social reward on response inhibition in children and adolescents. Developmental science 12(4): 614–625. 19635087 10.1111/j.1467-7687.2009.00816.x

[80] Milteer RM, Ginsburg KR (2012) The Importance of Play in Promoting Healthy Child Development and Maintaining Strong Parent-Child Bond: Focus on Children in Poverty. Pediatrics 129 (1): e204–e213.. 22201149 10.1542/peds.2011-2953

[81] Toplak ME, Tannock R (2005) Time perception: modality and duration effects in attention-deficit/hyperactivity disorder (ADHD). Journal of abnormal child psychology 33(5): 639–654. 16195956 10.1007/s10802-005-6743-6

[82] Toplak ME, Dockstader C, Tannock R (2006) Temporal information processing in ADHD: findings to date and new methods. Journal of neuroscience methods 151(1): 15–29. 16378641 10.1016/j.jneumeth.2005.09.018

[83] van Meel CS, Oosterlaan J, Heslenfeld DJ, Sergeant JA (2005) Motivational effects on motor timing in attention-deficit/hyperactivity disorder. Journal of the American Academy of Child & Adolescent Psychiatry 44(5): 451–460. 15843767 10.1097/01.chi.0000155326.22394.e6

[84] Sonuga-Barke E, Thompson M, Daley D, Laver-Bradbury C (2004) Parent training for Attention Deficit/Hyperactivity Disorder: Is it as effective when delivered as routine rather than as specialist care? British Journal of Clinical Psychology 43: 449–457. 15530214 10.1348/0144665042388973

[85] Sonuga-Barke E, Bitsakou P, Thompson M (2010) Beyond the dual pathway model: Evidence for the dissociation of timing, inhibitory, and delay-related impairments in attention-deficit/hyperactivity disorder. Journal of the American Academy of Child & Adolescent Psychiatry 49(4): 345–355. 20410727 10.1016/j.jaac.2009.12.018

[86] Pelham W (2010) 1-2-3 Magic. Effective discipline for children 2-12. Revised 4^th^ Edition. Quality books Inc. Illinois.

[87] Webster-Stratton C, Reid MJ, Beauchaine TP (2012) One-Year Follow-Up of Combined Parent and Child Intervention for Young Children with ADHD. Journal of Clinical Child and Adolescent of Consulting and Clinical Psychology 65: 93–109. 10.1080/15374416.2012.723263 PMC3584230 23020199

[88] Hill M, Duncan G (1987) Parental Family Income and the Socioeconomic Attainment of Children. Social Science Research 16(1): 39–73.

[89] Liddle EB, Hollis C, Batty MJ, Groom MJ, Totman JJ, et al (2011) Task-related default mode network modulation and inhibitory control in ADHD: effects of motivation and methylphenidate. J Child Psychol Psychiatry 52(7): 761–771. 21073458 10.1111/j.1469-7610.2010.02333.x PMC4754961

[90] Daley D, van der Oord S, Ferrin M, Danckaerts M, Doepfner M, et al.. (2014) Behavioral Interventions in Attention-Deficit/Hyperactivity Disorder: A Meta-Analysis of Randomized Controlled Trials across Multiple Outcome Domains. Journal of the American Academy of Child & Adolescent Psychiatry doi: 10.1016/j.jaac.2014.05.013 10.1016/j.jaac.2014.05.013 25062591

[91] Beck A, Daley D, Hastings RP, Stevenson J (2004) Mothers' expressed emotion towards children with and without intellectual disabilities. Journal of Intellectual Disability Research 48(7): 628–638. 15357682 10.1111/j.1365-2788.2003.00564.x

