# Supplementary figures and images for: Linguistic Analysis of the Preschool Five Minute Speech Sample: What the Parents of Preschool Children with Early Signs of ADHD Say and How They Say It?
Source: PLoS One. 2014 Sep 3;9(9):e106231. doi: 10.1371/journal.pone.0106231 (PMC4153579; doi:10.1371/journal.pone.0106231)

**Appendix S1**

**Clinical Data**


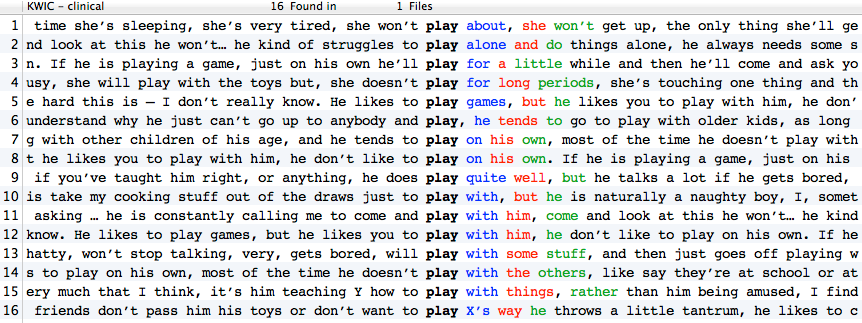

Supplement: Appendix S1 — (DOCX) [file pone.0106231.s001.docx]

**Appendix S2**

**Control data**


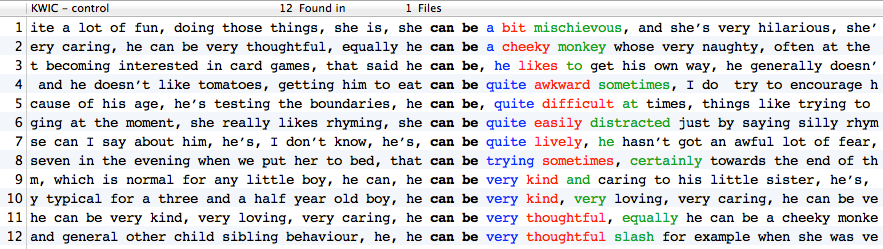


**Clinical data**


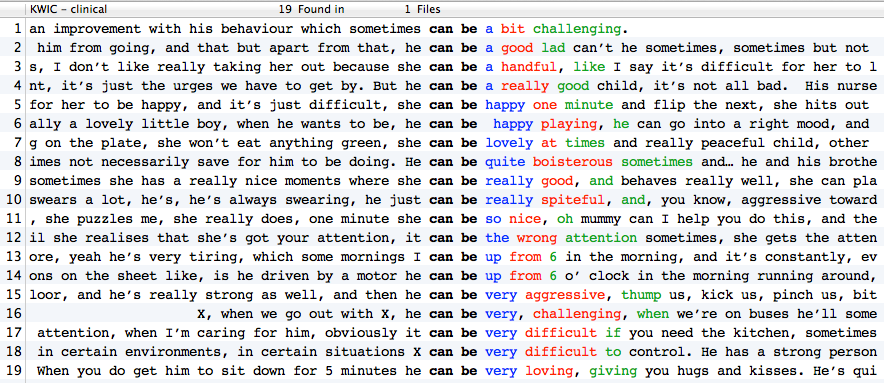

Supplement: Appendix S2 — (DOCX) [file pone.0106231.s002.docx]
